# Habitat characterization and spatial distribution of *Anopheles sp*. mosquito larvae in Dar es Salaam (Tanzania) during an extended dry period

**DOI:** 10.1186/1475-2875-4-4

**Published:** 2005-01-14

**Authors:** Michael A Sattler, Deo Mtasiwa, Michael Kiama, Zul Premji, Marcel Tanner, Gerry F Killeen, Christian Lengeler

**Affiliations:** 1 Swiss Tropical Institute, P.O. Box, 4002 Basel, Switzerland; 2 Ministry of Regional Administration and Local Government, Dar es Salaam, Tanzania; 3 Muhimbili University College of Health Sciences, Institute of Public Health, Dar es Salaam, Tanzania; 4 Ifakara Health Research and Development Center, P.O. Box 53, Ifakara, Kilombero, Morogoro, Tanzania

## Abstract

**Introduction:**

By 2030, more than 50% of the African population will live in urban areas. Controlling malaria reduces the disease burden and further improves economic development. As a complement to treated nets and prompt access to treatment, measures targeted against the larval stage of *Anopheles sp*. mosquitoes are a promising strategy for urban areas. However, a precise knowledge of the geographic location and potentially of ecological characteristics of breeding sites is of major importance for such interventions.

**Methods:**

In total 151 km^2 ^of central Dar es Salaam, the biggest city of Tanzania, were systematically searched for open mosquito breeding sites. Ecologic parameters, mosquito larvae density and geographic location were recorded for each site. Logistic regression analysis was used to determine the key ecological factors explaining the different densities of mosquito larvae.

**Results:**

A total of 405 potential open breeding sites were examined. Large drains, swamps and puddles were associated with no or low *Anopheles sp*. larvae density. The probability of *Anopheles sp*. larvae to be present was reduced when water was identified as "turbid". Small breeding sites were more commonly colonized by *Anopheles sp*. larvae. Further, *Anopheles gambiae s.l*. larvae were found in highly organically polluted habitats.

**Conclusions:**

Clear ecological characteristics of the breeding requirements of *Anopheles sp*. larvae could not be identified in this setting. Hence, every stagnant open water body, including very polluted ones, have to be considered as potential malaria vector breeding sites.

## Background

### Urban malaria

Urbanization is progressing fast worldwide. By the year 2030, more than 50% of the African population will live in urban areas [1]. It is anticipated that infectious disease problems including vector-borne diseases such as malaria might also increase in urban areas [2]. Keiser et al. [3] estimated that 24.8–103.2 million clinical malaria episodes occur annually in urban settings endemic for malaria. Urban malaria is generally characterized by: low transmission intensity, lack of immunity in the population and higher mortality rates in older age groups [4]. The distribution pattern of malaria transmission intensity is dependent on the degree of urbanization and on the distance from vector breeding sites [5,6]. Consequently, a high heterogeneity of transmission intensity is characteristic for urban malaria [7]. Increasing urban agriculture is thought to play a major role in increasing malaria in urban areas. However, this is not yet demonstrated, as it increases breeding sites for *Anopheles sp*. mosquito larvae, but it also raises the economic status of the population, allowing improved malaria protection [8].

Malaria is not only a personal health problem, but also an intolerable economic burden [9]. Investments in fighting malaria would result in substantial economic gains [10]. Therefore lowering the malaria burden in urban areas with a high economic development potential could result in improved economic development and serve as an example for other areas [7].

In urban areas, relatively few breeding sites are found per unit population [7]. In addition, the high human density usually allows a better access to curative and preventive health services. As a result, a number of malaria control options such as environmental control, including vegetation clearance, modification of river boundaries, draining swamps and insecticide application to open water bodies become possible and cost-effective [7,11,12]. With prompt access to antimalarials and other vector control interventions such as insecticide-treated bednets, integrated malaria control becomes realistic in African cities with a well developed municipal service delivery infrastructure [3,7,13].

### Mapping of breeding sites and mosquito ecology as basis for integrated malaria control

Every campaign to reduce malaria transmission aims to be as effective as possible given existing resources. Malaria control methods are affected by many setting-specific factors such as endemicity, vector species and behaviour, seasonality, disease patterns, health service factors and more. Since these factors are not distributed equally in space, accurate and timely information is required before malaria control can be planned and resources allocated properly. With regard to transmission reduction, attention must be paid to the areas of greatest vector abundance and precise risk maps of *Anopheles sp*. breeding sites are of major importance. Computerized maps have proven to be useful for this purpose, helping to understand malarial epidemiology and guiding interventions [14-18].

Besides geographic location, knowledge of ecological features of mosquito breeding sites is a potential key element for implementing efficient and effective larvae control measures. Such measures have been shown to be an important tool to reduce malaria endemicity [19,20]. In an urban environment *Anopheles sp*. mosquitoes adapt to new breeding sites created by urbanization, and hence their ecology might differ from rural settings [21]. Most African studies *on Anopheles sp*. mosquito larvae ecology have been conducted in rural settings [7,22], and findings from these studies might not be applicable to urban settings without adaptation.

The spatial distribution of *Anopheles sp*. and Culicine larvae in open breeding sites in Dar es Salaam, Tanzania, were examined during an extended dry period and correlations between biological and physicochemical environmental factors and the abundance of mosquito larvae were determined. This work was carried out in order to collect baseline information for a larval control-based mosquito abatement programme implemented by the three municipalities of Dar es Salaam.

## Materials and Methods

### Study site

**Figure 1:**
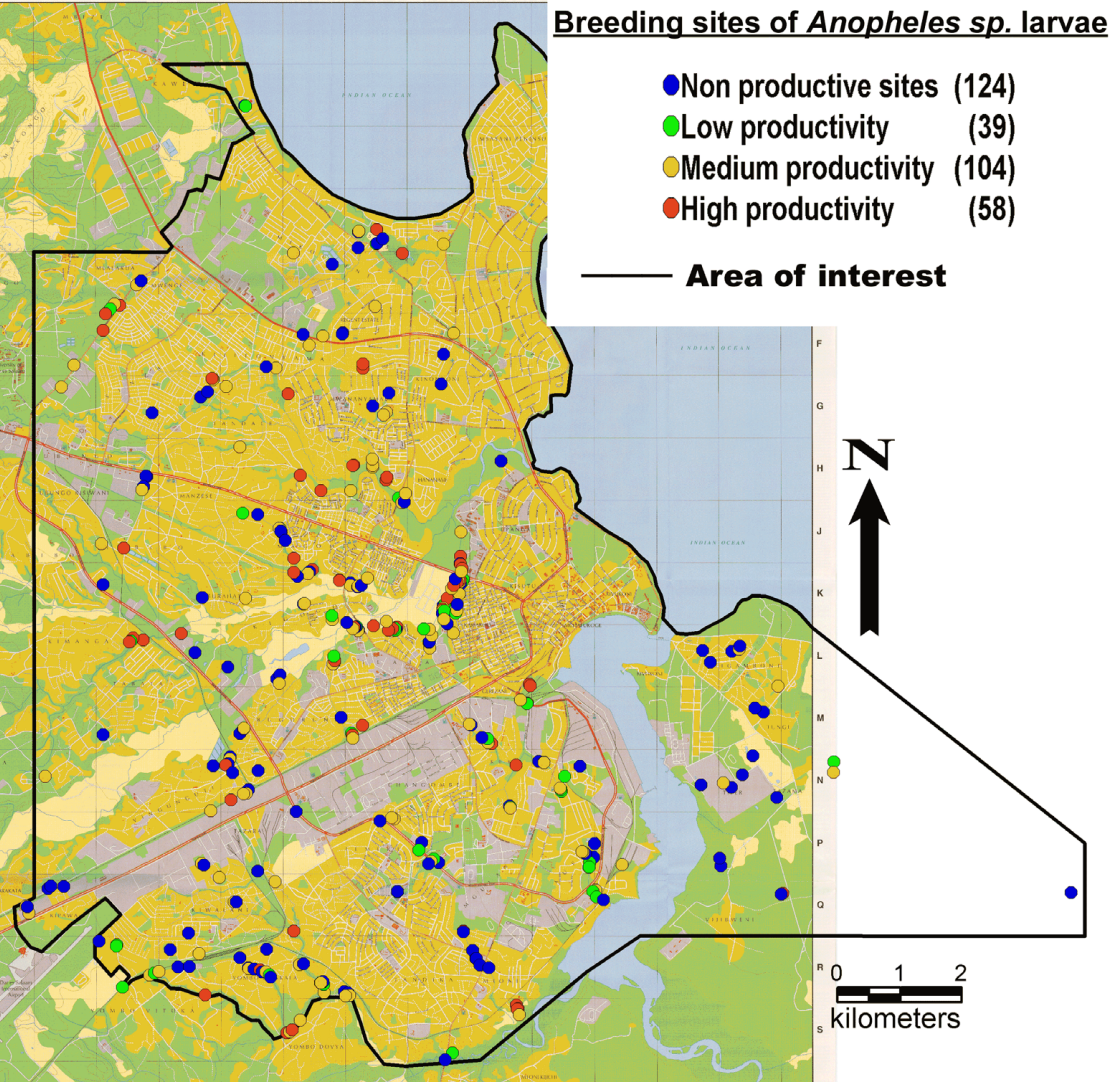
Breeding sites of *Anopheles sp*. larvae in Dar es Salaam, Tanzania.

Dar es Salaam is the biggest and economically most important city of Tanzania, with a population of more than three million people and an area of 1450 km^2^. The present study area covers 151 km^2^. in the central area of the city (Figure 1). Although highly urbanized, the study area is dotted with swamps, ponds and lakes and many additional water bodies are created for agricultural purposes. Combined with the hot and humid climate, this results in excellent breeding opportunities for *Anopheles sp*. mosquitoes. In Dar es Salaam the main malaria vectors are: *Anopheles gambiae s.s*., *Anopheles arabiensis*, *Anopheles funestus *and *Anopheles merus *[23-25].

### Study period

The data collection was conducted from March 1 to May 29, 2003. Even though the study period was meant to be during the rainy season, the actual rainfall (236 mm) was less than half the long-term average from 1961–1990 (576 mm) [26]. Furthermore, the previous short periods of rainfall also failed, so this represents part of an extended drought period. Each area was visited only once in a cross-sectional survey of breeding sites.

### Mosquito identification

To distinguish between *A. gambiae s.l*. (*gambiae s. s*., *arabiensis*, *merus*) and *A. funestus*, larvae collected from the productive breeding sites were raised and hatched in the laboratory so that the adults could be identified morphologically by light microscopy using the key of Gillies and De Meillon [27].

*Anopheles gambiae s.l*. were not further identified to sibling species by PCR because such a methodology is not practical, affordable or particularly useful in the context of routine operations of a sustainable mosquito abatement program in Dar es Salaam.

### Breeding site identification and recording of ecological parameters

Each ward within the study area was searched for open water bodies. To locate areas of potential breeding sites, the search was lead by municipal malaria experts with a good knowledge of the area. Furthermore, one-year-old geo-referenced digital aerial pictures (0.5 m ground resolution, produced by Geospace International, Pretoria, South Africa) were examined visually on the computer and all suspected open water areas were checked on the ground by extracting coordinates from the digital picture and locating the area in the field using a global positioning system (GPS) (eTrex^©^, Garmin International Inc., Olathe, US). In addition, 10 year-old breeding site maps from the Japan International Cooperation Agency (JICA) were consulted [24]. All open bodies of water were taken as potential breeding site and geo-referenced by GPS.

The presence of larvae at high or low densities was determined by dipping. From every potential breeding site up to 10 dips were taken with a standard white 350 ml dipper. If *Anopheles sp*. could be seen without dipping or nearly every dip contained *Anopheles sp*. larvae, the site was defined as having a high *Anopheles sp*. density. Sites where only one or two dips out of 10 contained *Anopheles sp*. larvae were defined as having a low *Anopheles sp*. density. Sites where no *Anopheles sp*. larvae could be found in ten dips were recorded as empty. Pupae were not recorded as they cannot be differentiated from non-*Anopheles *species in the field. To attempt to quantify the relative mosquito larvae productivity of each breeding site, a productivity score was assigned to each breeding site according to its size and mosquito larvae density (Table 1). Sites which were dry at the time of visit were excluded.

**Table 1:** Classification of larvae productivity in potential breeding sites

	Perimeter of site	Perimeter < 1 m	Perimeter 1–10 m	Perimeter > 10 m
			
Larval density				
Absent		0	0	0
Low		1	1	2
High		1	2	3

0 = No larvae productivity, 1 = Low larvae productivity, 2 = Medium larvae productivity 3 = High larvae productivity

The same definition was also used to characterize Culicine mosquitoes (mainly consisting of the genera *Culex*, *Aedes *and *Mansonia*) density in the same sites.

To record habitat type, every site was categorized as one of the following: stream, small drain, large drain, swamp, rock pool, puddle, footprints, tyre track, artificial hole, concrete hole, artificial container and other. Further biological and physicochemical factors were measured, as shown in Table 2. All visual classifications were done by the same person to maintain consistency. In addition, the general setting was qualitatively described, mentioning special features and general impressions.

**Table 2:** Key parameters measured in the 327 sites that contained water, Dar es Salaam, Tanzania.

**Characteristics**	#	%	**Characteristics**	#	%
**Type of breeding site:**			**Filamentous algae present**	138	42.2
Stream	2	0.6			
Large drain	29	8.9	**Single-celled algae present**	18	5.5
Small drain	16	4.9			
Swamp	92	28.1	**Predators present**	118	36.1
Rock pool	0	0			
Puddle	22	6.7	**Shade**		
Foot/hoof print	4	1.2	0–25%	250	76.5
Tyretrack	1	0.3	26–50%	58	17.7
Artificial hole	82	25.1	51–75%	16	4.9
Concrete hole	9	2.8	75–100%	3	0.9
Artificial container	0	0			
Other	69	21.4	**Maximum depth more than 0.5 m**	108	32.7
					
**General surrounding**			**Size**		
High density housing	236	71.6	Perimeter < 1m	17	5.2
Fields	57	18.0	Perimeter 1–10 m	128	39.4
Industry	19	5.8	Perimeter > 10 m	182	55.4
Other	15	4.6			
			**Turbidity**		
**Distance to nearest inhabited house**			Clear	137	41.9
<10 m	179	54.7	Turbid	160	48.9
10–100 m	127	38.8	Very turbid	30	9.2
>100 m	21	6.4			
			**Salinity mS/cm (rounded to full numbers)**		
**Distance to nearest potential resting site**			0–1	138	42.3
<10 m	327	100	2–3	145	44.5
10–100 m	0	0	4–5	22	6.7
>100 m	0	0	6–7	4	1.2
			8–9	3	0.9
***Anopheles *larvae density**			10–14	4	1.2
Absent	125	38.2	15–19	2	0.6
Low density	64	19.3	>20	8	2.5
High density	138	42.5			
			**Temperature °C (rounded to full numbers)**		
***Culicine *larvae density**			25–26	9	2.8
Absent	139	43.1	27–28	34	10.4
Low density	43	13.1	29–30	92	28.2
High density	145	43.7	31–32	71	21.7
			33–34	63	19.3
***Anopheles *and *Culicine *pupae density**			35–36	39	12.0
Absent	247	75.5	37–38	12	3.7
Low density	37	11.3	39–40	5	1.5
High density	43	13.1			
			**pH (rounded to full numbers)**		
**Grass in the middle of site present**	134	41.0	6	3	0.9
			7	111	34.1
**Grass along the edges present**	244	74.6	8	169	52.0
			9	46	12.3
**Floating plants present**	65	19.9	10	2	0.6
**Floating algae present**	41	12.5			

### Statistical analysis

Statistical analysis was carried out with SPSS V 11.0 (SPSS Inc., Chicago, US). Logistic regression analysis was used to determine the importance of key factors for explaining the different mosquito larvae densities. For each group of mosquitoes *(Anopheles sp*. and Culicines) two logistic regression models were fitted: one model was used to determine the key factors influencing whether larvae were present or absent, and one model was used to determine the key factors influencing whether larvae were present at low or high density. For the second model only data from sites that contained at least one larva were used. All measured parameters were included in the logistic models. The three categories "stream", "foot/hoofprint" and "tyre track" were included in the baseline category "other" as they had only four or less records. No "artificial containers" or "rockpools" were found, consequently these categories are not presented in the analysis. Salinity, temperature and pH were measured with an electronic device (HANNA^© ^HI 98130 Combo pH&EC, Hanna Instruments Inc., Kehl am Rhein, Germany). Conductivity measured in milliSiemens/cm was the indicator to measure salinity. The continuous variables "salinity" "temperature" and "pH" were categorized into quartiles for ease of interpretation. As this study is focused on *Anopheles sp*. larvae, only the significant parameters are presented for the Culicine models. To reduce the risk of α errors given the large number of variables that where investigated, only variables with p-values below 0.01, or those showing a consistent trend in both regression models with p-values below 0.05, were considered in the discussion.

### Ethics

This study did not involve human subjects and permission was obtained from the National Institute of Medical Research in Tanzania (NIMR/HQ/R.8a/Vol.lX/l 12, No. 2003-110-CC-2003-63).

## Results

### Open breeding sites in Dar es Salaam

**Figure 2:**
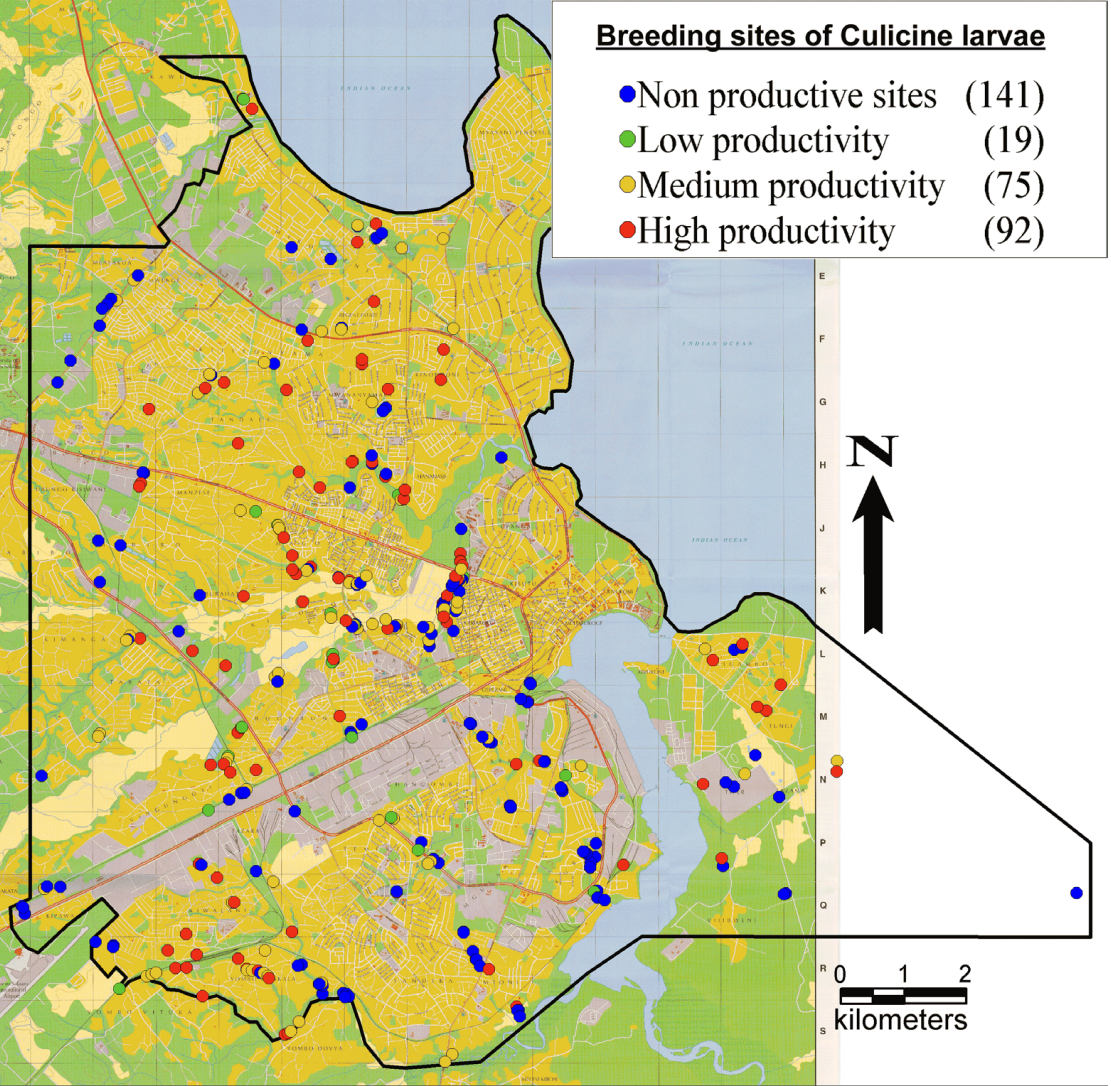
Breeding sites of Culicine larvae in Dar es Salaam, Tanzania.

A total of 405 potential mosquito breeding sites were examined and mapped. 19% were dry at the time of visit. Of the 327 sites that contained water, 62% were productive for *Anopheles sp*. and 57% were productive for Culicine mosquitoes. From all 150 adult mosquitoes reared from the larvae samples, only *A. gambiae s.l*. could be found. In one productive breeding site salinity was above 1%, suggesting that these larvae were *Anopheles merus *[28]. The 202 sites productive for *Anopheles sp*. were made up of 69% low larvae density sites and 31% high larvae density sites. Of the 186 sites productive for Culicine larvae 23% had low larvae density and 77% had high larvae density. Breeding sites of *Anopheles *sp. and Culicines and their productivity are shown in Figures 1 and 2.

### Key factors determining the presence/absence or low/high density of mosquito larvae

The frequencies of the different parameters are shown in Table 2.

*Presence/absence of Anopheles sp. larvae *(Table 3): Large drains, swamps and puddles were much less likely to contain *Anopheles sp*. larvae than other habitats. Very turbid water diminished the chance that *Anopheles sp*. larvae were present. Breeding sites with a size of less than one meter were more likely to contain *Anopheles sp*. larvae.

**Table 3:** Output of logistic regression model with presence versus absence of *Anopheles sp*. larvae as outcome and ecological parameters as explanatory variables.

**Variable**		**Odds Ratio**	**95% Confidence Interval for OR**		**P-Value**
			**Lower**	**Upper**	
**Type of breeding site**	Other	1			
	Large drain	.145	.046	.459	.001**
	Small drain	.492	.111	2.188	.352
	Swamp	.139	.055	.346	.000**
	Puddle	.196	.051	.752	.018*
	Artificial hole	.481	.186	1.245	.132
	Concrete hole	1.407	.156	12.702	.761
**General surrounding**					
	High density housing	1			.118
	Other	.812	.210	3.131	.762
	Industry	.211	.055	.808	.023*
	Fields	.502	.207	1.216	.127
**Distance to nearest inhabited house**					
	<10 m	1			
	10–100 m	1.889	.473	7.541	.368
	>100 m	1.839	.940	3.596	.075
***Culicine *density**					
	high	1			
	low	.856	.369	1.988	.718
	absent	.687	.356	1.328	.265
**Size of site**					
	Perimeter > 10 m	1			
	Perimeter 1–10 m	1.932	.928	4.020	.078
	Perimeter < 1 m	22.825	2.094	248.843	.009**
**Turbidity of water**					
	clear	1			
	turbid	.469	.231	.954	.037*
	very turbid	.167	.059	.473	.001**
**Salinity measured by conductivity [mS/cm] (Quartiles)**					
	2.6–20.0	1			
	1.7–2.5	.522	.228	1.195	.124
	1.1–1.6	1.418	.576	3.490	.447
	0.1–1.0	.728	.313	1.696	.462
**Temperature (°C) (Quartiles)**					
	25.1–29.2	1			
	29.3–31.3	1.758	.676	4.571	.247
	31.4–33.4	.731	.306	1.744	.480
	33.5–40.0	1.148	.507	2.597	.741
**pH of water (Quartiles)**	8.1–10.1	1			
	7.7–8.0	1.773	.711	4.426	.220
	7.4–7.6	.825	.330	2.059	.680
	6.1–7.3	.439	.180	1.070	.070
Predators present		1.931	1.031	3.619	.040*
Sun-lit (less than 50% of surface shaded)		1.913	.575	6.363	.290
Depth >0.5 m		.750	.393	1.434	.384
Grass present		.952	.601	1.508	.834
Floating vegetation present		.935	.570	1.534	.790
Submersed vegetation present		1.201	.679	2.124	.529

* = p < 0.05 ** = p < 0.01

*High/low density of Anopheles sp. larvae *(Table 4): When *Anopheles sp*. larvae were present in large drains, swamps and puddles, these sites were much less likely to contain high densities of *Anopheles sp*. larvae.

**Table 4:** Output of logistic regression model with high versus low *Anopheles sp*. larvaedensity as outcome and ecological parameters as explanatory variable.

**Variable**		**Odds Ratio**	**95% Confidence Interval for OR**		**P-Value**
			**Lower**	**Upper**	
**Type of breeding site**					
	other	1			
	large drain	.124	.021	.739	.022*
	small drain	.768	.109	5.389	.791
	swamp	.182	.053	.629	.007**
	puddle	.165	.033	.832	.029*
	artificial hole	.712	.198	2.556	.602
	concrete hole	.348	.032	3.727	.383
**General surrounding**					
	high density housing	1			
	other	.358	.056	2.290	.278
	industry	2.675	.326	21.950	.359
	fields	.925	.275	3.112	.899
**Distance to nearest inhabited house**					
	<10 m	1			
	10–100 m	.658	.261	1.656	.374
	>100 m	.409	.054	3.069	.384
***Cuttcine *density**					
	high density	1			
	low density	.728	.221	2.396	.602
	absent	1.078	.432	2.688	.872
**Size of site**					
	perimeter > 10 m	1			
	perimeter 1–10 m	1.565	.576	4.255	.380
	perimeter < 1 m	.907	.166	4.950	.911
**Turbidity of water**					
	clear water	1			
	turbid water	1.653	.692	3.951	.258
	very turbid water	2.116	.372	12.030	.398
**Salinity measured by conductivity [mS/cm] (Quartiles)**					
	2.7–20.0	1			
	1.7–2.6	1.638	.479	5.599	.431
	1.2–1.6	.818	.251	2.671	.740
	0.1–1.1	.687	.227	2.079	.507
**Temperature (°C)(Quartiles)**					
	34.1–40.0	1			
	31.6–34.0	1.004	.315	3.203	.994
	29.4–31.5	1.312	.356	4.830	.683
	25.1–29.3	.619	.176	2.184	.456
**pH of water (Quartiles)**					
	8.2–10.1	1			
	7.8–8.1	.882	.228	3.419	.856
	7.5–7.7	.777	.200	3.027	.716
	6.5–7.4	.793	.198	3.182	.744
**Predators present**		1.322	.530	3.302	.550
**Sun-lit (less than 50% of surface shaded)**		1.035	.184	5.832	.969
**Depth >0.5 m**		.460	.174	1.219	.118
**Grass present**		.801	.272	2.362	.688
**Floating vegetation present**		.352	.130	.952	.040*
**Submersed vegetation present**		1.745	.725	4.197	.214

* = p < 0.05 ** = p < 0.01

*Presence/absence of Culicine larvae *(Table 5a): Turbid water was clearly associated with the presence of Culicines. Breeding sites with pH values of 7.6 or less were also more likely to contain Culicine larvae.

**Table 5:** Output from a logistic regression model with (a) presence versus absence of *Culicine *and (b) high versus low density of *Culicine *larvae as outcome, and ecological parameters as explanatory variable. The same variables as in tables 2 and 3 were included in the model, but only significant results are shown.

5a					
**Variable**		**Odds Ratio**	**95% Confidence Interval for OR**		**P-Value**
			**Lower**	**Upper**	

**General Surrounding**					
	High density housing	1			
	Other	.646	.170	2.453	.520
	Industry	.177	.041	.771	.021*
	Fields	.692	.302	1.585	.384
**Turbidity of water**					
	Clear	1			
	Turbid	3.620	1.872	7.000	.000**
	Very turbid	2.689	.981	7.369	.054
					
**pH of water (Quartiles)**					
	8.1–10.1	1			
	7.7–8.0	1.986	.845	4.669	.116
	7.4–7.6	2.916	1.252	6.792	.013*
	6.1–7.3	2.586	1.109	6.030	.028*

5b					

**Variable**		**Odds Ratio**	**95% Confidence Interval for OR**		**P-Value**
			**Lower**	**Upper**	

**Type of breeding site**					
	Other	1			
	Large drain	4.207	.575	30.801	.157
	Small drain	.836	.054	12.894	.898
	Swamp	1.811	.507	6.467	.361
	Puddle	2.797	.345	22.650	.335
	Artificial hole	1.137	.288	4.486	.855
	Concrete hole	.059	.005	.733	.028*
**pH of water (Quartiles)**					
	8.0–9.0	1			
	7.6–7.9	2.291	.748	7.014	.146
	7.4–7.5	2.485	.707	8.740	.156
	6.4–7.3	4.024	1.230	13.158	.021*

*High/low density of Culicine larvae *(Table 5b): A pH value of less than 7.3 was associated with high Culicine larvae density.

### Productive *Anopheles sp*. *gambiae s.l*. mosquito breeding sites in Dar es Salaam: unexpected results

**Figure 3:**
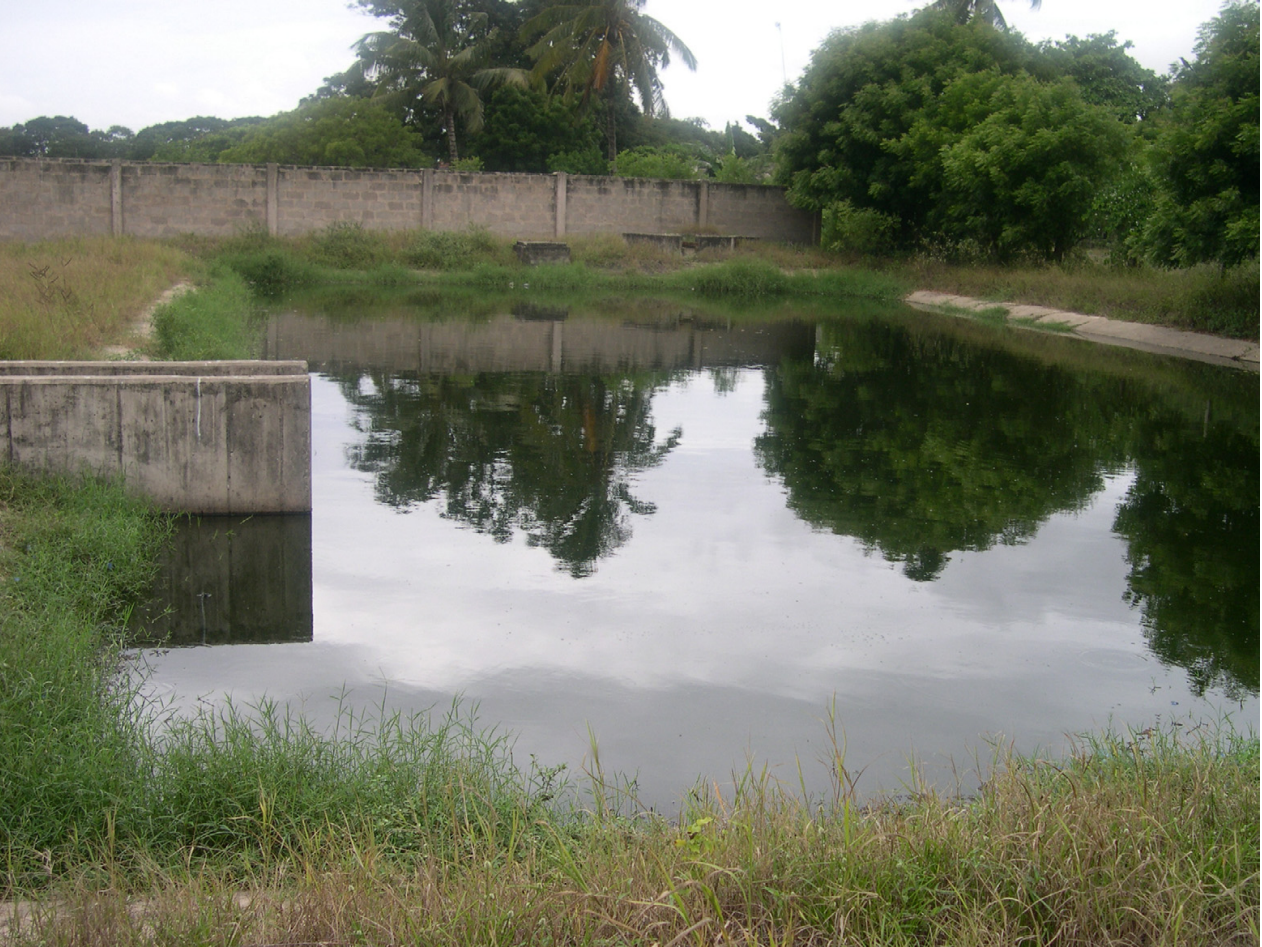
Sewage pond in Temeke municipality, Dar es Salaam, Tanzania.

**Figure 4:**
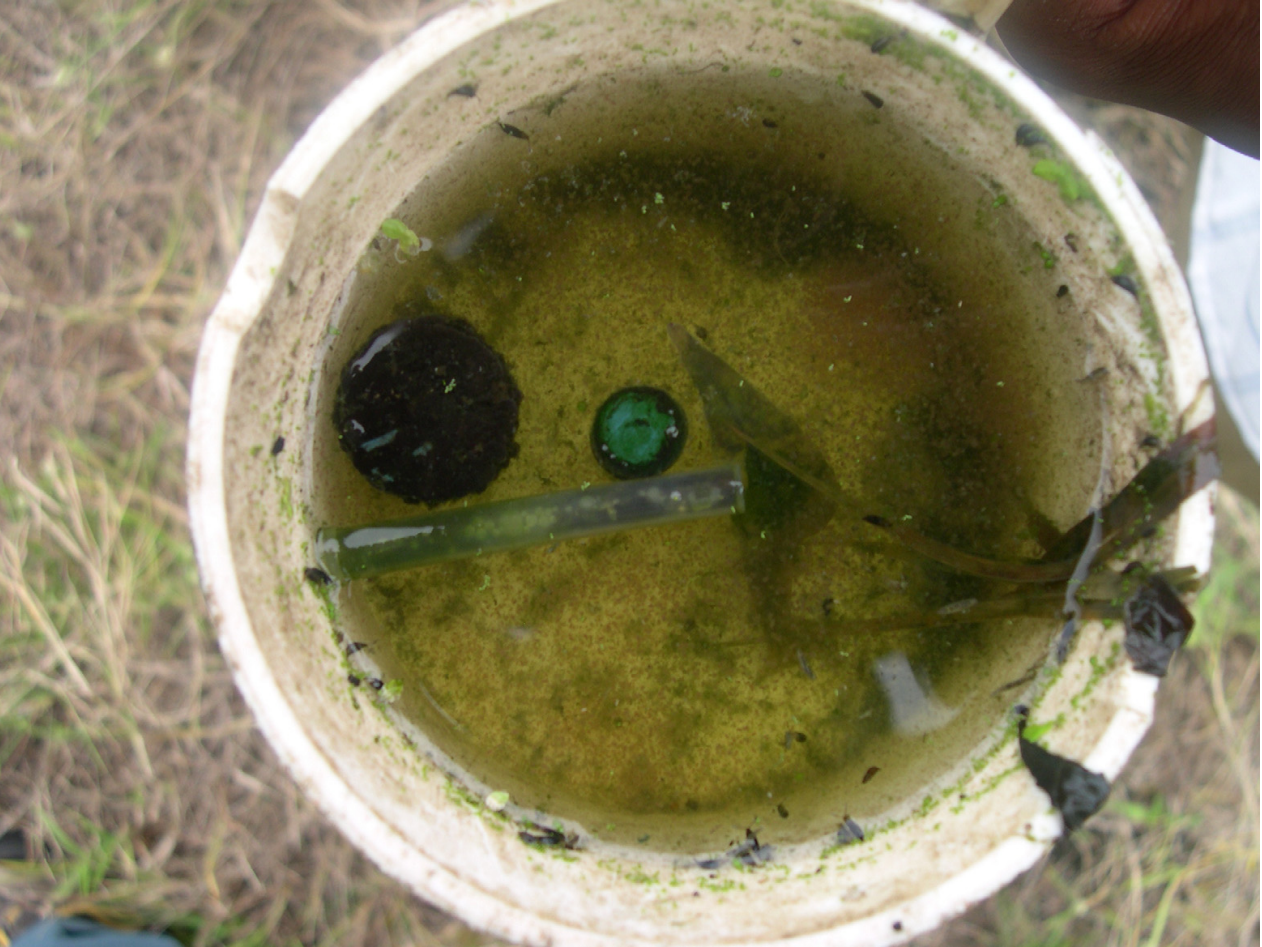
Dipper content from sewage pond in Temeke municipality (Figure 3).

Contrary to the conventional thinking that *A. gambiae s.l*. only breeds in rather clean and clear water [28,29], such larvae were found in habitats organically polluted by rotting vegetation, human faeces or oil. Such sites were the drain of an oil refinery, one organically polluted swamp used as garbage dumping area and one sewage pond with organic pollution from human faeces (Figures 3 and 4). Except for the breeding site type "tyretrack", for which only one potential habitat was identified, *An. gambiae s.l*. could be found in all types of habitats recorded during the study.

### Socio- and geo-ecological environments with high mosquito breeding site density

**Figure 5:**
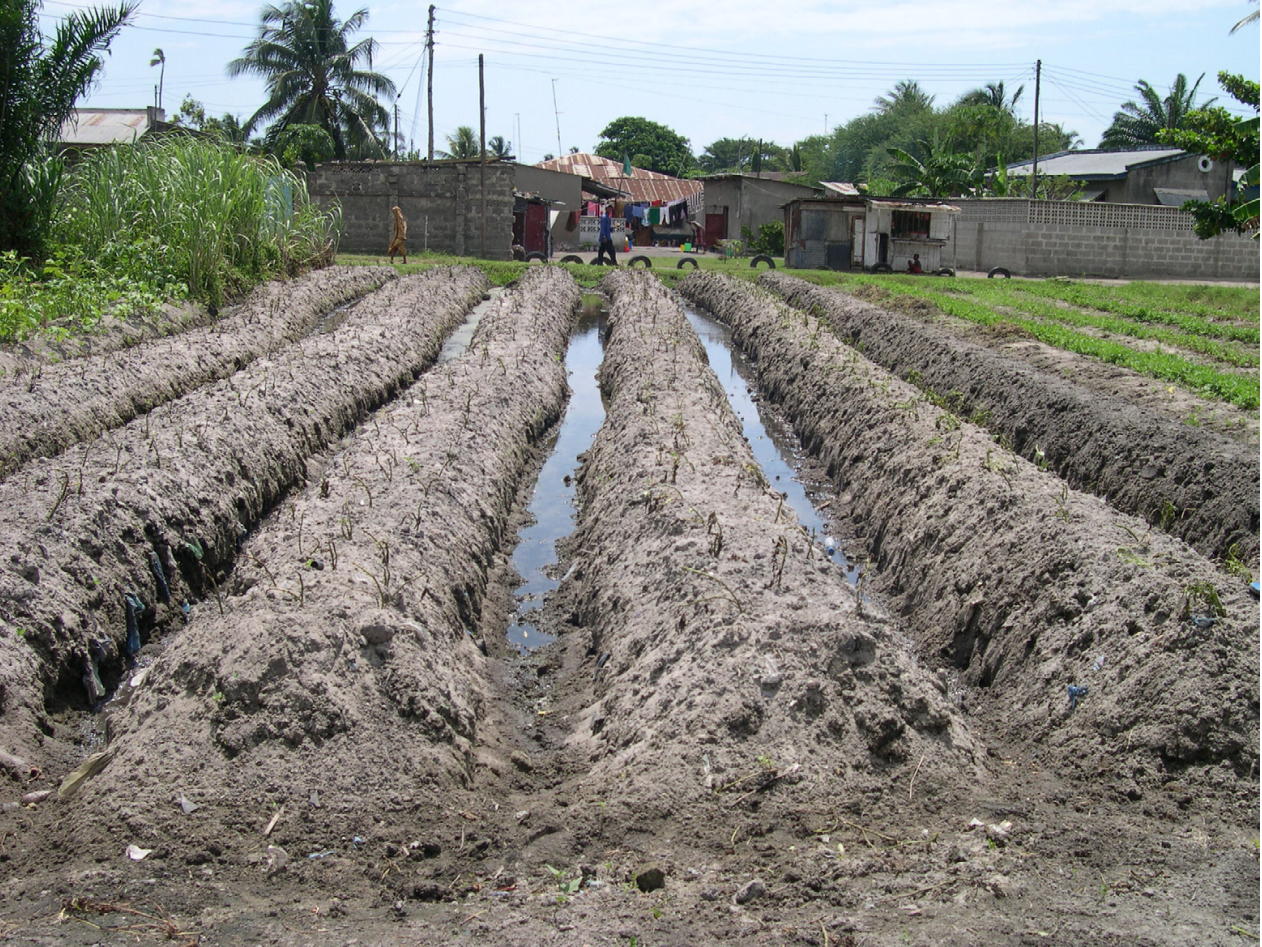
"Matuta" type of agriculture in Dar es Salaam, Tanzania.

In our study six environments were defined where larval control interventions are of high priority due to the large number of highly productive breeding sites for *Anopheles sp*. Most environments identified as potentially suitable for mosquito breeding in Dar es Salaam could be described by a close interaction between geo-ecological settings and the influence of humans. Slopes on riverbeds, riverbeds, borders of swamps, stagnant drains and rivers, areas with restricted access and sites along railway lines made up the six high productivity environments (for further details see: Sattler [30]). As Dar es Salaam is highly populated, houses have been built nearly everywhere. Only swampy areas, riverbeds and steep slopes are not, or only sparsely, populated due to the danger of flooding or landslides. Further, housing is forbidden on military property or other restricted areas such as the international airport. All areas defined as potentially productive have a very high water table, as found in most parts of Dar es Salaam. As a result, these areas represent also suitable land for small-scale farming. The type of agriculture using "*matuta*" is most likely to lead to mosquito breeding. Matuta is a type of agriculture where plants are grown on top of little ridges, while between the ridges deep furrows are dug. These furrows are often filled with a little water, forming shallow pools perfectly suitable for *Anopheles sp*. breeding (Figure 5). Further, rice fields, shallow wells and irrigation channels were also found to be very productive for *Anopheles sp*. in the focus areas.

## Discussion

### Main limitations of the study

This study represents a snapshot of a highly dynamic system and presents the first results of a larger malaria control program. More intensive longitudinal studies are currently being undertaken to complement these results.

The study was implemented during an exceptionally dry year and, although the study took place during the rainy season, it actually rained very little [26]. As a result, it is unclear whether the breeding site structure found during the three months of our sampling period was representative of the structure found in years with normal rainfall. Similarly, the measured ecological parameters might be different during more normal years, as the selectivity of mosquitoes for oviposition sites can be greatly diminished during dry periods because of the limited availability of aquatic habitats [27]. In years with normal rainfall, it is very likely that many habitats that contained larvae during the study period would be unproductive due to a flushing effect, while other small productive sites might appear. However, for the purpose of reducing mosquito density by larvae abatement, the time when the mosquito population is most vulnerable is the dry period. *A. gambiae s.s*., *A. arabiensis *and *A. funestus *survive during the dry period in discreet habitats, making them an easier target for control interventions [31]. Dry season larval control is for example the rule in South Africa [32].

Very small breeding sites could not be detected by aerial pictures and were, therefore, largely excluded from the study. This could result in the omission of factors potentially important for mosquito control interventions. Further, mosquitoes do not necessarily lay eggs every day in each potential breeding site, thus the reason why a site did not contain larvae could simply be because no eggs were deposited within the last week.

One of the key objectives of the study was to detect ecological factors determining *Anopheles sp*. larvae productivity. But within a city, the influence of humans can never be neglected. The pollution from waste such as oil, soap or industrial by-products is important in potential breeding sites in Dar es Salaam. Larvae may have been absent due to pollution by elements that could not be detected in the frame of this study.

*Anopheles sp*. mosquitoes were not classified down to species level. The goal of the study was to characterize important breeding sites of *Anopheles sp*. mosquitoes and, consequently, potential foci of malaria transmission, regardless of the species. This is because in the context of sustainable operations in a routine mosquito abatement programme, municipal staff cannot be expected to identify all *Anopheles *larvae samples to species level without rendering sampling procedures prohibitively laborious and expensive. To achieve a satisfactory impact, exhaustive targeting of all potential vector species is necessary anyway. Furthermore, community acceptance of vector control programmes in Dar es Salaam has been shown to require suppression of all mosquito species, rather than only malaria vectors [25].

### Ecological factors influencing mosquito larvae density

The abundance of *Anopheles sp*. and Culicine larvae seems to be influenced differentially by ecological parameters, as none of them had a significant effect in all four regression models. Only one factor influenced the presence or absence of both *Anopheles sp*. and Culicine larvae. In turbid breeding sites Culicine larvae were much more likely to be present, whereas *Anopheles sp*. larvae were much more likely to be absent. Bates [29] supports this finding of *Anopheles sp*. breeding in rather clear water, but Gimnig *et al*. [33] found increasing *A. gambiae s.l*. larvae densities with increasing turbidity. Robert *et al*. [34] found a clear water preference by *A. ambiensis *breeding in wells in urban Dakar. A study by Ye-Ebiyo *et al*. [35] found that the production of *A. arabiensis *was favoured in moderately turbid water, while excessive turbidity limited the production of larvae. The proximity to flowering maize with pollen as food source compensated for the development failure induced by excessive turbidity. Clearly, the simple definition of "turbidity" might not be precise enough. Water which is turbid from particles not edible for *Anopheles sp*. larvae could disfavour the production of larvae, while water turbid from food particles represents a very suitable habitat. The preference of Culicine mosquitoes for turbid water is coherent with their known breeding site preferences, as they breed successfully in rather polluted environments such as blocked drains and septic tanks [36,37].

### Key factors influencing the density of *Anopheles sp*. larvae

In small breeding sites with diameters less than 1 m, *Anopheles sp*. were more likely to be present than in larger habitats. This is consistent with the general description of breeding sites for *A. gambiae s.l*., but not with those for *A. funestus *[27,36]. However, this finding could be biased by the fact that rain was lacking for several weeks, evaporating the bigger pools and concentrating the larvae, hence making them easier to detect. Further, larval density of small breeding sites might be increased due to a higher sampling intensity per unit area.

*Anopheles *larvae were less likely to be present in swamps, and when so, they were found in low densities. This finding matches the known preference of *A. gambiae s.l*. for temporary sites [36,38]. Even though swamps were less likely to harbour *Anopheles sp*., the importance of these habitats should not be underestimated because of their great size and their role in supplying water for irrigation ditches, rice fields and various agricultural activities.

"Puddle" as a type of breeding site was not favoured by *A. gambiae s.l*. larvae, and when *Anopheles sp*. larvae were present there, they were likely to be present in low densities. This finding is not consistent with established habitat descriptions for *Anopheles sp*. larvae [25,39].

Large drains were likely to have low *Anopheles sp*. densities. This result agrees with findings by Yamagata [24]. Large drains were often organically polluted with waste water and, therefore, less suitable for *Anopheles sp*. breeding [21,28,38].

### Key factors only influencing the density of Culicine larvae

A pH value of less than 7.3 favoured high Culicine densities and a value of less than 7.6 favoured their presence. These results showed clearly that Culicine larvae favoured a pH-neutral environment.

### *Anopheles sp*. breeding sites: unexpected findings

The findings of this study revealed that *A. gambiae s.l*. was found in a sewage pond (Figure 3 and 4) and in one swamp extremely polluted with organic matter. These findings from Dar es Salaam, together with other studies, could indicate a change of *Anopheles sp*. breeding requirements in urban settings. In Lahore, Pakistan, *Anopheles sp*. mosquitoes were found in the waste water system [40]. In Accra, Ghana, data collections since 1911 indicate that *A. gambiae s.l*. adapted to breeding in organically polluted water habitats [21]. As most studies of *Anopheles sp*. larval ecology have been conducted in rural settings, it is likely that unpolluted breeding sites are found and described far more often [7,22], giving a biased impression. One of the main problems is the term "polluted habitat", as it has never been clearly defined. The findings of this study show that *Anopheles sp*. larvae can breed in nearly every kind of water accumulation. For every ecological factor identified as enhancing or reducing *Anopheles sp*. larvae productivity, at least one breeding site was found that contradicted these findings. Hence, all water bodies in an urban environment should be considered as potential breeding places and a target for larval control.

### Defined socio- and geo-ecological environments with high mosquito breeding sites density

Slopes to riverbeds, riverbeds, borders of swamps, stagnant drains and rivers, areas with restricted access and areas along railway lines represented environments where most breeding sites were found in Dar es Salaam. These sites where mostly associated with agriculture activities. Afrane *et al*. [8] stated that areas of high mosquito density tended to follow valleys, where breeding sites were most persistent. Also, in Brazaville the valleys with vegetable gardens and crops were identified as some of the most suitable places for *Anopheles sp*. breeding [5]. In Dakar, one big marshy area was responsible for the production of nearly all adult *Anopheles sp*. mosquitoes within a distance of one kilometer [6]. Similar results were found by Staedke *et al*. [41]. Ponds close to the embankments of a railway line are presented as potential dry-season refugia for mosquito by Charlwood *et al*. [31].

The increasing urbanization of Dar es Salaam will probably help to reduce risk areas in the long-term, as open spaces get rare and swampy areas are filled to regain building land, but unplanned city growth along the city edges could increase the number of breeding sites [22]. In Brazaville, areas with the lowest malaria transmission intensity corresponded to the oldest and most densely populated districts [5]. Furthermore, high density housing has been shown to reduce breeding sites more than medium-density housing in Kisumu and Malindi, Kenya [22].

## Conclusions

Mapping of malaria risk on the basis of breeding sites plays an important role for urban malaria control programs. Also, initial risk mapping of breeding sites, combined with improved knowledge of mosquito ecology and their interactions with humans, is crucial to understand the epidemiology of urban malaria. The present study, in accordance with other studies of *Anopheles sp*. on urban larval ecology, showed that *Anopheles sp*. mosquito larvae are not restricted to clearly defined habitats. Therefore, malaria control interventions, such as environmental measures or insecticide application, have to consider all open water bodies as potential breeding sites. Specific malaria control interventions are currently developed and tested by the three municipalities of Dar es Salaam.

## Authors' contributions

MS designed and implemented the study, analyzed the data and drafted the manuscript. DM, MK and ZP were involved in designing and implementing the study and analyzing its data. MT participated in the study design, data analysis and drafting of the manuscript. GK and CL assisted in the study design and implementation, data analysis and writing of the manuscript. All authors read and approved the final manuscript.
